# Snaq: A Dynamic Snakemake Pipeline for Microbiome Data Analysis With QIIME2

**DOI:** 10.3389/fbinf.2022.893933

**Published:** 2022-07-01

**Authors:** Attayeb Mohsen, Yi-An Chen, Rodolfo S. Allendes Osorio, Chihiro Higuchi, Kenji Mizuguchi

**Affiliations:** ^1^ Artificial Intelligence Center for Health and Biomedical Research (ArCHER), National Institutes of Biomedical Innovation, Health and Nutrition, Osaka, Japan; ^2^ Institute for Protein Research, Osaka University, Osaka, Japan

**Keywords:** snakemake, QIIME2, microbiome, 16S, automation

## Abstract

Optimizing and automating a protocol for 16S microbiome data analysis with QIIME2 is a challenging task. It involves a multi-step process, and multiple parameters and options that need to be tested and determined. In this article, we describe Snaq, a snakemake pipeline that helps automate and optimize 16S data analysis using QIIME2. Snaq offers an informative file naming system and automatically performs the analysis of a data set by downloading and installing the required databases and classifiers, all through a single command-line instruction. It works natively on Linux and Mac and on Windows through the use of containers, and is potentially extendable by adding new rules. This pipeline will substantially reduce the efforts in sending commands and prevent the confusion caused by the accumulation of analysis results due to testing multiple parameters.

## Introduction

The microbial content of a biological sample can be determined by sequencing and the subsequent bioinformatic processing/analysis of the sequenced data. 16S Ribosomal RNA gene sequencing (Hugerth and Andersson, 2017) is one of the most intensively used approaches in microbiome research. It is also called amplicon sequencing since it incorporates the amplification of a specific DNA region (16S rRNA gene) in bacterial genomes using PCR. Accordingly, software tools such as QIIME 2 (Bolyen et al., 2019) and Mothur (Schloss et al., 2009), to name only two, have been developed for the processing and analysis of this type of data. For a detailed description of 16S amplicon approach please refer to (Gołębiewski and Tretyn, 2019) and for a comparison between tools that can be used for its analysis, we recommend (Bolyen et al., 2019; Prodan et al., 2020).

QIIME2 (Bolyen et al., 2019) is a microbiome data analysis platform that targets amplicon (16S) data. It relies on third party software programs implemented as plugins (such as feature-classifier (Bokulich N. A. et al., 2018) for taxonomic classification), QIIME2 is designed to facilitate seamless incorporation of new plugins, allowing developers to add new features easily^1^.

QIIME2 plugins handle input and output through the definition of *artifacts*, i.e., compressed folders that contain both data files and metadata information. For example, raw sequence data can be imported to construct an artifact, which is later used by a specific plugin. In turn, the plugin produces a new artifact of a different type as output.

This approach makes it possible to change the order of the steps or insert new steps in the middle without extra effort, provided that the input and output follow the QIIME2 framework guidelines. This approach makes combining multiple tools in sequence effortless and reduces the requirements of programming skills. Moreover, importing or exporting data from/to various formats or visualization of the original data or the results can be achieved easily.

Despite its multiple advantages, some difficulties arise when trying to automate data analysis using QIIME2. Even when the same data types and the same experimental technique (such as sample preparation proceduce or sequencing technology) are used, the results of the analysis depend on multiple environmental and technical features, such as the length and quality of the sequenced data. As the choice of the bioinformatics tools and processing parameters (such as quality trimming threshold or sequence similarity) used for the analysis depend on the data set, every data set can be considered unique and in need of special treatment, making it impossible (or very difficult) to automate.

For example, the process of *quality trimming* is used to clean the data and improve the results by removing the nucleotides assigned with low confidence. Selecting the appropriate quality threshold value at which trimming should be performed depends on the data set, and usually requires trying and testing. If a very stringent trimming threshold is adopted, plenty of good data could be lost; alternatively, adopting a loose trimming threshold introduces low-quality data in the downstream analysis, affecting the quality and reliability of the results. Moreover, there are different tools and databases for data cleaning, identifying Operational Taxonomy Units (OTUs), and taxonomy assignment. The choice of these tools will affect the final result, and hence fine tuning is required in order to find the best options.

Usually, researchers need to investigate multiple options, which requires running the analysis several times and comparing the results to decide the best set of tools and parameters for the data under investigation. Such an optimization process requires a substantial effort and leads to the accumulation of several copies of the results with different sets of parameters, making the whole process rather inefficient and difficult to reproduce.

By combining the analysis strengths of QIIME2 with the flexibility in the definition of pipelines provided by Snakemake, here we introduce “Snaq”, a dynamic Snakemake pipeline for microbiome data analysis with QIIME2.

Snaq incorporates the definition of analysis rules with the definition of an expressive target file format, which together provide the functionality required to achieve the following when working with QIIME2:

1) Faster protocol optimization: By changing the name of the target file, the analysis workflow dynamically changes, allowing testing of different tools and parameters. This is crucial, as the analysis of 16S microbiome data with QIIME2 can be performed in multiple ways with numerous permutations of software and parameter choices, depending on the technology used and sequencing qualities, and the researcher’s preference.

2) Full pipeline automation: Combining rule definition with an ad-hoc target file name, Snaq allows the execution of a full analysis pipeline through a single command instruction. As 16S microbiome data analysis with QIIME2 entails multiple command submission, this significantly reduces the number of commands and instructions that the user needs to know, allowing to focus on the actual analysis and not the programming.

3) Handle data accumulation: Snaq automatically handles the (intermediate) data that are often generated as a result of multiple trial runs. Additionally, it avoids the duplication of intermediate result files when multiple executions of different analysis pipelines include identical intermediate stages.

## Related Work

Various tools and efforts have been developed to make QIIME 2 more accessible and easy to use.

Fung et al. introduced the QIIME2 automation pipeline (QAP), a series of scripts that could be used to run multiple QIIME2 protocols (Fung et al., 2021). In addition, their paper gives detailed explanations of many steps and descriptions of their results. Multiple commands need to be executed to run the analysis using QAP; moreover, it provides more options and different approaches than Snaq follows (Fung et al., 2021).

Estaki et al. (2020) provided a comprehensive description of QIIME2. They also, with the help of Jupyter notebooks, provide examples of running end to end analysis using QIIME2.

In an effort closer to Snaq, Hu and Alexander implemented a Snakemake pipeline for QIIME2 analysis, designed to run with parameters specified through a configuration file (Hu and Alexander, 2020). Due to its design, the change of parameters requires the modification of manifest and configuration files. Additionaly, tasks like trimming and taxonomy assignment are not covered.

Dadasnake is another example of a Snakemake pipeline that automates DADA2 analysis outside the setting of the QIIME2 framework (Weißbecker et al., 2020).

Also worth mentioning at this point is the Galaxy project, an open-source platform that allows users to do data analysis within the FAIR initiative (Afgan et al., 2018). Included in its directory of tools is q2Galaxy, a comprehensive interface for QIIME2 https://github.com/qiime2/q2galaxy . q2Galaxy makes performing microbiome data analysis easier especially when docker is used for its installation.

Although the above mentioned tools are available and help automate the analysis of 16S data, none of them (with the only exception of q2Galaxy) provides an easy way to run an analysis multiple times as is usually required for optimization purposes. Also, they tend to have fixed steps and/or do not make it easy to change the sequence of steps and parameters used.

To address this issues, we would like to propose a pipeline that make it easy to modify the key parameters used by different tools, by simply modifying the target file name. We also expect our single command approach to make it easier to run analysis multiple times without the user having to worry about the handling or intermediate output results.

## Implementation

Snakemake (Koster and Rahmann, 2012; Mölder et al., 2021) is a Python dialect created for the specification of pipeline workflows. A Snakemake pipeline is specified through the definition of *rules*; where each rule typically has: *input* for the specification of input files; *output* for the specification of output files; and *shell* for the specification of the command used to produce the output based on the input.

The execution of a Snakemake pipeline is achieved via the definition of a single target file name. Snakemake will then determine the steps required to produce the target output based on its rules, the file name, and the application of the wildcards concept^2^.

The wildcards concept facilitates passing parameters for any rule in the pipeline by inferring the parameter’s value from the target file name. This feature of Snakemake is especially suitable for parameter optimization. There are other advanced features, such as caching processing results, to prevent doing the same analysis repeatedly (Koster and Rahmann, 2012).

Snaq is made of three main components: 1) Snakefile, 2) env folder and 3) scripts folder. Snakefile is the file where all the required snakemake rules are implemented; notice these rules were carefully constructed as not to contend with each other and to make the whole process run smoothly. The env folder contains the definitions for the Conda (Anaconda, 2020) environments as a series of YAML files, while the scripts folder contains extra scripts required by Snaq to fill the gaps of the pipeline that are not covered by QIIME2 plugins.

Snaq takes advantage of QIIME2’s command-line interface and available plugins and combines it with our implementation of new Python and R scrips. Then, by incorporating a descriptive name file convention and the rule-based structure of Snakemake, it makes possible the definition and execution of dynamically defined pipelines through a single terminal command.

Snaq can be used on personal computers or server environments. It works on Linux and Mac operating systems^3^. It is also possible to use directly from the available Docker and singularity containers. All analysis takes place in the Snaq home folder, where the input files need to be stored inside data folder, and all results will be saved in results folder.

### Descriptive File Convention

**FIGURE 1:**
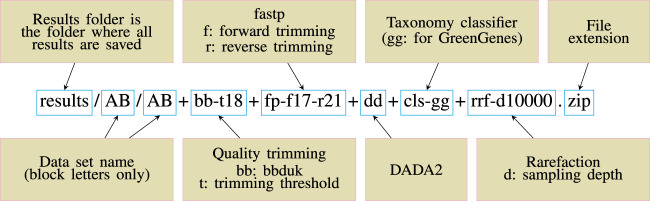
Descriptive target file name example. A target file is required for Snaq to execute correctly.

To make the pipeline versatile and easily modifiable, we adopted a convention of including all the key parameter values inside a target file name and called this scheme descriptive target file naming (Figure: 1). At the same time, other parameters are left as default. This means that Snakemake will parse the target file name and infer the sequence of steps and the parameter values used. Then the target file will be created accordingly.

To let Snakemake infer the required steps and their order, we used a predetermined output nomenclature for each stage (Table 1).

**TABLE 1:** Output nomenclature.

Term	Explanation
fp-f{x}-r{y}	fp: stands for fastp application, x:takes the number of nucleotides to be cropped from forward read, y: takes the value of the number of nucleotides to be cropped from reverse read
bb-t{t}	bb: stands for bbduk application, t: is the trimming threshold applied
dd	dd: stands for DADA2 algorithm
cls-{x}	cls: stands for taxonomy classifier, x: takes one of the values: “gg” for Greengenes, “silva” for SILVA classifier and “silvaV34” for SILVA classifier trained on V3 and V4 regions
rrf-d{x}	rrf: stands for rarefaction and x is the value of the rarefaction
alphadiversity	alpha diversity
beta	beta: stands for beta diversity

The stages of analysis in the target file name are divided using the character “+” (plus sign). For example, let us consider the case of the target file shown in Figure 1. Here, we are requesting Snaq to produce a summarized result (indicated by the extension .zip) for the input data located in folder data/AB/ ^4^. Sequentially, bb-t18 indicates a trimming stage with threshold value 18; fp-f17-r21 indicates the use of fastp with a forward cropping value of 17 and a reverse cropping value of 21; dd indicates the use of the DADA2 algorithm; cls-gg request a taxonomy classification using Greengenes; and finally rrf-d10000 indicates the use of rarefaction with a sampling depth of 10,000. It is worth noting that the order of the analysis follows the order of stages.

We believe that in most cases, users will use Snaq through the definition of a single target file and a single command-line instruction. However, intermediate results files can also be produced upon request by using the corresponding target file. For example, if a user were only to import the dataset into a QIIME2 artifact, this could be done by using results/AB/AB.qza as target file name. Similarly, if trimming were to be added, the corresponding target file would be results/AB/AB+bb-t18.qza, and so on. Notice that the addition of stages is typically in a forward fashion; this means that later analysis stages can not be added to the target file name without their previous stages also being part of it.

**FIGURE 2:**
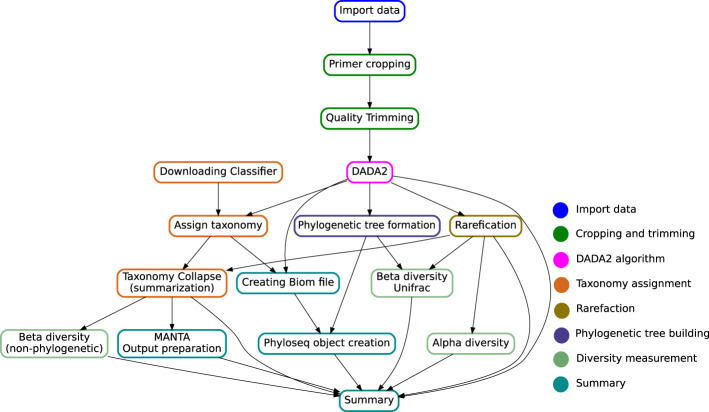
Directed Acyclic Graph showing the key steps in the Snaq pipeline. Notice that the given target file will determine a specific traversal of the graph. Color of nodes is used to indicate the analysis stage, as proposed in the implementation section.

The multiple stages specified in the target file name define an execution pipeline, as the one shown in Figure 2.

In the following, we provide a detailed description of the pipeline stages and their corresponding^5^ descriptive file conventions:

• Import data: This stage imports FASTQ files from the source folder to a QIIME2 artifact (qza). Notice that, to avoid any confusion, dataset needs to be named using only capital letters. The results for this step are stored in folder results/AB/AB.qza. The command required to run this step is: snakemake ––use-conda ––cores 10 results/AB/AB.qza Hereafter, we will omit the command and options (snakemake ––use-conda ––cores 10), and focus only on the target file name.
• Primer cropping: This stage uses fastp (Chen et al., 2018) to crop a specified number of nucleotides in both reads. The format of the target is fp-fX-rY where X represents the number of nucleotides to be cropped from the 5′ end of the forward reads (R1), and Y is the number of nucleotides to be cropped from the reverse reads (R2). For example, in order to add the cropping of 17 bases from the 5′ end of R1 and 21 bases from R2 to our previously loaded dataset, the target would be: results/AB/AB+fp-f17-r21.qza
• Quality trimming: It uses bbduk (part of the bbmap tools) (Bushnell, 2021) to trim the section with low quality at the end of the reads in both R1 and R2. The format target for this step is bb-tX where X represents the trimming threshold. To add a quality trimming of reads with threshold of 18, the target file name becomes:
results/AB/AB+fp-f17-r21+bb-t18.qza
• Both primer cropping and quality trimming procedures are optional (can be omitted) and their order can be reversed. For example, the following target file names are also valid:
results/AB/AB+bb-t18.qza
results/AB/AB+bb-t18+fp-f17-r21.qza
• DADA2 algorithm: The DADA2 (Callahan et al., 2016) stage filters the reads, joins pairs, and removes chimera producing Amplicon sequence variant tables (ASVs) that replace OTUs in traditional clustering methods such as UCLUST (Callahan et al., 2017). As result, three different outputs are generated: an Amplicon sequence variant (ASV) frequency table (dd_table.qza), a table of representative sequences for ASVs (dd_seq.qza) and the statistics of DADA2 performance (dd_stats.qza). Using any one of these targets will trigger the generation of all three files, for example:
results/AB/AB+fp-f17-r21+bb-t18+dd_seq.qza
  When the DADA2 algorithm stage is part of a longer pipeline, its inclusion in the target file name can be simply identified by using the word dd (see next section’s target file name).
• Taxonomy assignment: It uses the “feature-classifier” plugin (Bokulich NA. et al., 2018; Robeson et al., 2021) to predict the taxonomy of ASVs. Three different classifiers are available: Greengenes (cls-gg) (DeSantis et al., 2006; McDonald et al., 2011), SILVA (cls-silva) (Pruesse et al., 2007; Glöckner et al., 2017), and SILVA trained on V3 and V4 regions (cls-silvaV34) (Mohsen, 2021). The resulting output can be generated both as a QIIME2 artifact (cls-<classifier>_taxonomy.qza) or as tab separated file (cls-<classifier>_taxonomy.tsv). For the Greengenes classifier, the two target file name alternatives would be:
results/AB/AB+fp-f17-r21+bb-t18+dd+cls-gg_taxonomy.qza and results/AB/AB+fp-f17-r21+bb-t18+dd+cls-gg_taxonomy.tsv
• Phylogenetic tree building: This step uses the fasttree algorithm (Price et al., 2010) and QIIME2 phylogeny plugin (qiime2, 2021) to produce a phylogenetic tree file in NWK format (using fasttree.nwk as target) or QIIME2 artifact (using fasttree_rooted.qza as target). Notice that, since the building of a phylogenetic tree can be done directly after the DADA2 algorithm, the following is a valid target file name: results/AB/AB+bb-t18+fp-f17-r21+dd+fasttree.nwk
• Rarefaction: The inclusion of a rarefication stage is indicated by using the rrf-dX target, where X represents the sampling depth as defined in (Hughes and Hellmann, 2005). Notice that rarefaction needs to be applied before alpha diversity, non-phylogenic beta diversity measurements or the generation of biom tables. To apply rarefaction the following target file names can be used to generate a QIIME2 artifact, or a tab separated value file respectively:
results/AB/AB+bb-t18+fp-f17-r21+dd_table+rrf-d10000.qza
results/AB/AB+bb-t18+fp-f17-r21+dd_table+rrf-d10000.tsv
  In this step, the part _table was not omitted because rarefaction affects only the table and because in the following stages, this rarefied table is to be used to create biom tables and manta files.
• Diversity measurement: At this stage, QIIME2 is used for the computation of alpha (simpson, chao1, shannon and observed features) and beta diversities. Whilst the target for alpha diversity is simply alphadiversity, different types of beta diversity are specified through the target beta_<type>, where <type> is one of the following: braycurtis, jaccard, unweightedunifrac or weightedunifrac. Sample target file names are as follows:
results/AB/AB+bb-t18+fp-f17-r21+dd+rrf-d10000+alphadiversity.tsv
results/AB/AB+bb-t18+fp-f17-r21+dd+cls-gg+rrf-d10000+beta_braycurtis.tsv
results/AB/AB+bb-t18+fp-f17-r21+dd+cls-gg+rrf-d10000+beta_weightedunifrac.qza
  Notice that additional alpha and beta diversity measures can be added by modifying the scripts that define them and that can be found inside the scripts folder of Snaq.
• Summary: Having in mind the need of users to link the analysis made on snaq to other software tools, we prepared a series of special targets that generate results ready to be used elsewhere:
  • Phyloseq: Generates a Phyloseq object in RDS file, that can be easily imported to an R environment for subsequent analysis steps. This object includes the ASV table, taxonomy, and phylogenetic tree without rarefaction.
  • Biom: Produces biom table with taxonomy after rarefaction.
  • Manta: produces manta ready input files that can be easily uploaded in Manta for results storage and further analysis.
    Finally, a special zip file can be produced, as the one shown in Figure 1, that includes all content summarized in Table 2. A complete list of the files produced for an example analysis process is provided in Supplementary Table S1.
• Quality control: An additional, optional step, that runs fastqc (Andrews, 2010) and/or multiqc (Ewels et al., 2016). Unlike with other targets, the results of this stage are saved to a different quality folder, in our example: result/AB/quality. It is executed by using targets in the following form: results/AB/quality/AB+bb-t18/multiqc/results/AB/quality/AB/multiqc/
  Quality control can be applied either to the original data AB, or to any intermediate result obtained before the use of the DADA2 step. A special case that combines all fastqc reports in all subfolders of quality folder into a new quality_summary folder is achieved by using the target results/AB/quality_summary/.

**TABLE 2:** Summarized output, all the files are preceded by the parameters for the analysis.

Group	File name	Content
DADA2	cls-gg_taxonomy.tsv	tsv file of taxonomy assigned to ASVs
	table+rrf-d10000.tsv	Rarefied DADA2 features table
	dd_seq.tsv	ASV sequences produced by DADA2
Phyloseq	phyloseq.RDS	R phyloseq package object of data on ASV level
MANTA	manta.tsv	Taxonomy and abundance output friendly to be uploaded to a MANTA database
	manta_tax.tsv	Manta taxonomy ID and taxonomy names
Biom	otu_tax.biom	OTU BIOM table of the output collapsed to species level (ASV ignored)
	otu_tax_biom.tsv	OTU BIOM table saved as tsv
Diversity	alphadiversity.tsv	Table of alpha diversity for all samples
	beta_braycurtis.tsv	Braycurtis beta diversity
	beta_jaccard.tsv	Jaccard beta diversity
	beta_unweightedunifrac.tsv	Unweighted Unifrac distance between samples
	beta_weightedunifrac.tsv	Weighted Unifrac distance between samples

### Installation

**FIGURE 3:**
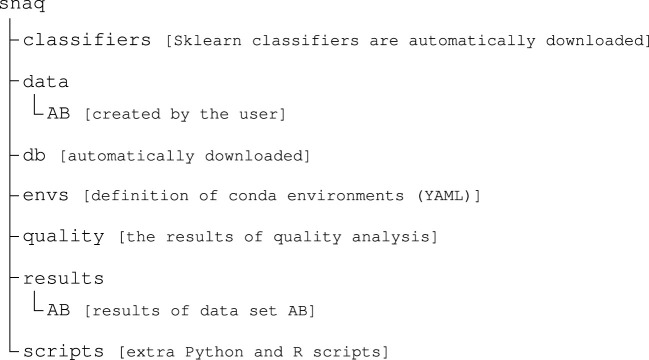
Folder structure of Snaq after installation (classifiers and quality folders will be created at later stage if required). Contents of data and results folders will vary according to use after installation. Notice that all data sub-folders need to be named using capital letters.

The only prerequisite on Linux or Mac is the installation of both Conda (Anaconda, 2020) and Mamba (QuantStack Scientific Computing, 2021). They are required to manage running environment, and facilitate the installation of QIIME2 and other required tools automatically. We recommend running Snaq after activating the Snakemake environment installed using Conda. Docker installation is the only requirement in running Snaq on Windows using a docker container. Figure 3 shows the file structure after installation.

The input data of Snaq are paired-end FASTQ files. Snaq automatically distinguishes pair ends by one of two identifiers _R1_ or _1.fastq. If other identifiers are used, a manifest file needs to be prepared and saved as results/AB/AB_manifest.tsv following the QIIME2 manifest file instructions. If this file is present, the first step of creating a manifest file will be ignored.

Other classifiers can also be added to the classifiers/folder, and the file name can be used in the target file name; if a classifier is named “abc” and saved as abc-classifier.qza, then we can use it with this target: results/AB/AB+bb-t18+fp-f17-r21+dd+cls-abc+rrf-d1000.zip without any modifications.

## Results and Discussion

Input data must be saved inside the data folder <snaq folder> /data/after creating a new folder with the dataset name inside it. Dataset names should consist of capital letters without numbers or special characters in order to avoid confusing them with terms reserved to represent different pipeline stages. Once input data is available and the first step in the analysis is executed, Snaq (through Snakemake) will automatically build the Conda environment required for that step and download the QIIME2 plugins specified in the corresponding environment YAML file. Environment description files are located in the <snaq folder>/envs/folder. Notice that this makes the installation of necessary software and the download of taxonomy classifiers an automatic process, only to be performed the first time it is required.

Although Snaq does not cover all the possible uses of QIIME2 and related platforms in 16S data analysis, it provides a complete pipeline that can be extended by adding new rules or modifying the currently available ones. Moreover, following the descriptive target file name strategy makes it easier for Snaq to decide which step to run and skip. That also gives the developer who wants to modify Snaq the freedom to modify the pipeline and add new rules besides the current ones, as a different sequence of rules can be followed depending on the target file name.

Compared to the pipelines mentioned above, Snaq allows dynamic modification of key parameters by modifying the target file name. It also provides a more straightforward installation process and clear output. Moreover, Snaq allows running multiple data sets in the same pipeline setting by having multiple folders in the data folder.

The concept of a descriptive output file name allows high freedom for the pipeline extension. New tools are added to the pipeline through the addition of new rules in the Snakefile. For example, to add trimmomatic the user simply requires to add the corresponding rule. For each new tool added to Snaq, the user requires to assign unique identifier, and identify any key parameters used by the tool. The identifier and parameters are then used in the definition of the Snakefile rule in order to specify the target that will later be used in the definition of an execution pipeline^6^.

Following on our example for trimmomatic, let us use tm as identifier and consider the use of a single parameter. Then, the rule in the Snakefile would follow a structure as shown in Code:1, whilst an example target name could be results/AB/AB+tm-p12+bb-t18, where tm identefies trimmomatic and p12 represents the specification of a value for its parameter.

rule NAME:
input:
“input-file”, “other-input-file"
output:
“<previous-step>+tm-pvalue.qza"
…
shell: <command> input output.

Code 1. Pseudo-code of a rule used for the incorporation of trimmomatic to Snaq.

## Conclusion

We have introduced Snaq, a Snakemake pipeline for QIIME2 16S data analysis, including data QC and trimming.

Snaq is designed to wrap QIIME2 processing of paired-end FASTQ files generated by Illumina sequencers to help automation, optimization, and take care of the data storage. It requires minimal effort in installation and configuration; moreover, it can run on all major operating systems. The user only needs a single command to run the pipeline defining required parameters for the analysis in the target file name.

Snaq can be installed directly from GitHub into a user specified location of choice. Notice that the installation directory needs to have enough free space to allocate for all input and intermediate data sets, together with all final results for any particular analysis. Free space is also required for the software programs and databases used in the analysis.

Snaq is designed to be dynamic by using a customly specified target file naming system. Modifying key parameters within the target name also helps the user efficiently perform a series of iterative analyses, taking automatic advantage of previously calculated intermediate steps and keeping track of results.

Installation and running of Snaq are easy. Moreover, Snaq can be extended according to the users’ needs by adding new rules.

## Data Availability

Publicly available datasets were analyzed in this study. This data can be found here: https://github.com/attayeb/snaq.

## Footnotes

1 For details on the architecture of QIIME2 visit https://dev.qiime2.org/latest/architecture/. Last accessed: 24 January 2022.

2 If a rule named “all” is defined, it is possible to run a predefined workflow without the specification of a target file.

3 Some tools required by QIIME2 do not run natively on Windows environments. Windows users could potentially use snaq through containers, windows Linux subsystem, and/or virtual machines.

4 Even when the command indicates the location of the result files, this also automatically maps to input folder under data/with the given name.

5 All examples assume the use of an input dataset, located in a source folder named data/AB folder.

6 Details for Snakemake rule definition can be found at https://snakemake.readthedocs.io/en/stable.
