# Supplementary material for: Snaq: A Dynamic Snakemake Pipeline for Microbiome Data Analysis With QIIME2
Source: Front Bioinform. 2022 Jul 1;2:893933. doi: 10.3389/fbinf.2022.893933 (PMC9580898; doi:10.3389/fbinf.2022.893933)
Supplement: Supplementary file 1 [file DataSheet1.PDF]

stage	file name
import data	AB_manifest.tsv<br>AB.qza
quality trimming	AB+bb-t18.qza
primer cropping	AB+bb-t18+fp-f17-r21.qza
	AB+bb-t18+fp-f17-r21+dd_seq.qza<br>AB+bb-t18+fp-f17-r21+dd_seq.tsv<br>AB+bb-t18+fp-f17-r21+dd_stats.qza<br>AB+bb-t18+fp-f17-r21+dd_table.qza
taxonomy assignment	AB+bb-t18+fp-f17-r21+dd+cls-gg_taxonomy.qza<br>AB+bb-t18+fp-f17-r21+dd+cls-gg_taxonomy.tsv<br>AB+bb-t18+fp-f17-r21+dd+cls-gg_asv.biom
phylogeny tree	AB+bb-t18+fp-f17-r21+dd+fasttree.nwk<br>AB+bb-t18+fp-f17-r21+dd+fasttree_rooted.qza<br>tree/
phyloseq	AB+bb-t18+fp-f17-r21+dd+cls-gg+phyloseq.RDS
rarefaction	AB+bb-t18+fp-f17-r21+dd_table+rrf-d10000.qza<br>AB+bb-t18+fp-f17-r21+dd_table+rrf-d10000.tsv
diversitry	AB+bb-t18+fp-f17-r21+dd+rrf-d10000+alphadiversity.tsv<br>AB+bb-t18+fp-f17-r21+dd+cls-gg+rrf-d10000+beta_braycurtis.tsv<br>AB+bb-t18+fp-f17-r21+dd+cls-gg+rrf-d10000+beta_jaccard.tsv<br>AB+bb-t18+fp-f17-r21+dd+rrf-d10000+beta_unweightedunifrac.qza<br>AB+bb-t18+fp-f17-r21+dd+rrf-d10000+beta_unweightedunifrac.tsv<br>AB+bb-t18+fp-f17-r21+dd+rrf-d10000+beta_weightedunifrac.qza<br>AB+bb-t18+fp-f17-r21+dd+rrf-d10000+beta_weightedunifrac.tsv
manta	AB+bb-t18+fp-f17-r21+dd+cls-gg+rrf-d10000+manta_tax.tsv<br>AB+bb-t18+fp-f17-r21+dd+cls-gg+rrf-d10000+manta.tsv
biom	AB+bb-t18+fp-f17-r21+dd+cls-gg+rrf-d10000+otu_tax.biom<br>AB+bb-t18+fp-f17-r21+dd+cls-gg+rrf-d10000+otu_tax_biom.tsv<br>AB+bb-t18+fp-f17-r21+dd+cls-gg+rrf-d10000+otu_tax.qza
summary	AB+bb-t18+fp-f17-r21+dd+cls-gg+rrf-d10000.zip

Supplementary Table 1: a complete list of all produced files for an imaginary data set “AB”.
